# Inferior Flap Tympanoplasty: A Novel Technique for Anterior Perforation Closure

**DOI:** 10.1155/2013/758598

**Published:** 2013-08-13

**Authors:** Haim Gavriel, Ephraim Eviatar

**Affiliations:** ^1^Department of Otolaryngology Head and Neck Surgery, Assaf Harofeh Medical Center, Zerifin 70300, Israel; ^2^Sackler Faculty of Medicine, Tel Aviv University, Ramat Aviv 69978, Israel

## Abstract

*Objective*. To report a novel tympanoplasty modification for anterior tympanic membrane perforation closure. *Materials and Methods*. A prospective study on 13 patients who underwent inferior tympanoplasty between December 2008 and May 2011 was carried out. In our technique, an inferior rather than a posterior flap is raised and the graft is laid from the inferior direction to obtain better access to the anterior part of the tympanic membrane perforation and provide better support. *Results*. A total of 13 patients underwent the novel inferior tympanoplasty technique with a mean age of 33 years. Six patients had undergone tympanoplasties and/or mastoidectomies in the past, 3 in the contralateral ear. A marginal perforation was observed in 3 cases, total perforation in 2 and subtotal in 1 case. The mean preoperative pure-tone average was 40.4 dB (10 to 90 dB), compared to 26.5 dB (10 to 55 dB) postoperatively. All perforations were found to be closed but one (92.3% success rate). *Conclusions*. The inferior tympanoplasty technique provides a favorable outcome in terms of tympanic membrane closure and hearing improvement for anterior perforations, even in difficult and complex cases. It is based on a well-known technique and is easy to implement.

## 1. Introduction

Tympanoplasty is an operative procedure used in the reconstruction of a perforation of the tympanic membrane (TM). Three main approaches are used in tympanoplasty: transcanal, endaural, and postauricular, and two classic methods for reconstruction of a TM perforation have been used: the underlay or overlay graft techniques. Each of these approaches and techniques has its advantages and disadvantages [1, 2].

The most common area of tympanoplasty failure when repairing total perforations is the anterosuperior area for several reasons including lack of graft support and less vascularity, with a greater risk of reperforation [3]. Added to these cases are marginal perforations, causing the closure rates to drop even further. Additionally, in many cases canaloplasty needs to be performed to remove the prominent anterior canal wall in order to visualize the entire perforation. Various surgical techniques have been reported to overcome these problems when treating anterior perforations, including “loop overlay” tympanoplasty [4], sandwich graft tympanoplasty [5], over-under tympanoplasty [6], mediolateral graft tympanoplasty [7], and others.

We present a novel modification of the traditional approach using an inferior rather than a posterior technique and report very high success rates, even in patients needing revision surgery due to reperforation after traditional tympanoplasty for closure of an anterior perforation. 

## 2. Materials and Methods

The study was approved by our Institutional Review Board (Institutional board approval no. 81/08).

A prospective review of consecutive patients with anterior tympanic membrane perforation undergoing inferior tympanoplasty between December 2008 and May 2011 was carried out. Demographic, surgical, and outcome data of these patients were collected. Middle Ear Risk Index was calculated for each patient. Included were those with anterior perforations of all sizes, including subtotal and total perforations. The procedure was not performed on patients requiring mastoidectomy for chronic otitis media with cholesteatoma or in patients with draining ears during a period of 3 months prior to the operation.

A total of 13 patients were included in the study. Each patient underwent preoperative audiological evaluation including speech reception threshold and speech discrimination testing.

*Surgical Technique.* The procedure is usually performed under general anesthesia. In our study, we used temporalis fascia grafts in all patients. 

A typical postauricular approach is performed to access the external ear canal and to obtain an appropriate temporal fascia graft. The edges of the perforation are freshened.

**Figure 1:**
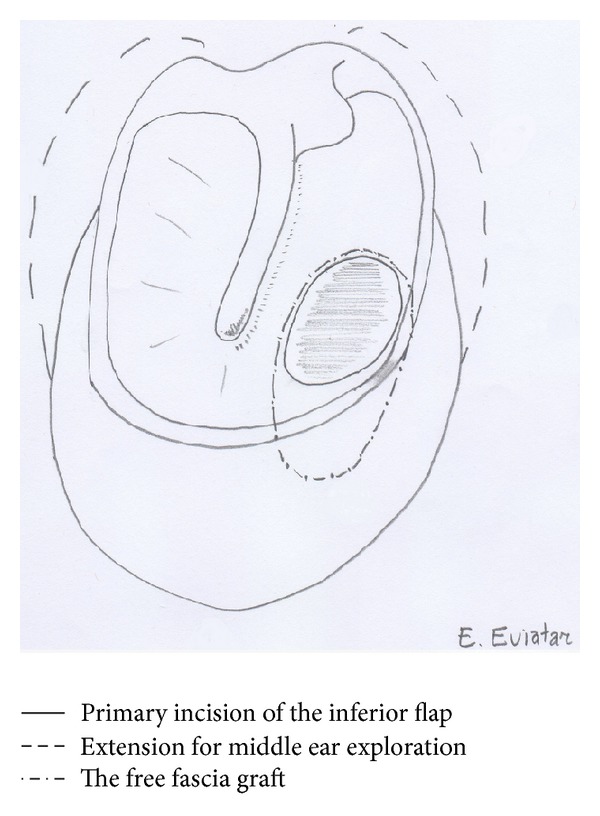
Illustration of the inferior tympanoplasty technique, right ear.

A transverse incision is created at the inferior aspect of the external auditory canal, curving medially gradually towards the 9 o'clock position anteriorly and the 3 o'clock position posteriorly on the left-hand side as shown in Figure 1 (3 o'clock position anteriorly and the 9 o'clock position posteriorly on the right-hand side). In cases with ossicular involvement, the posterior incision is extended further posteriorly towards the 12 o'clock position to expose the ossicular chain. An inferior tympanomeatal flap is raised. The annulus fibrosus is elevated from the annulus using a Rosen needle. Canaloplasty is usually not required in the postauricular approach but can be considered when visualization of the entire perforation is not possible. The middle ear space is assessed for any disease and, if present, it is managed. The ear is irrigated. The graft is then placed from the inferior direction superiorly to cover the anterior perforation, and a support is created medially and laterally using gel foam.

## 3. Results

A total of 13 patients underwent the inferior tympanoplasty technique, 5 males and 8 females with a mean age of 33 years (range 13–69). There were 6 left ears and 7 right ears. Six patients had undergone ear operation in the past, including tympanoplasties and mastoidectomies, 3 in the contralateral ear. One patient presented with bilateral tympanic perforation. All patients suffered from chronic otitis media without cholesteatoma. Four patients had a CT scan prior to the operation demonstrating a normal ear in 2 cases and middle ear effusion and mastoid sclerosis in 1 case each. A marginal perforation was observed in 3 cases, a total perforation in 2 and a subtotal in 1 case, while the rest had central anterior perforations. The mean MERI in our cohort was 2.69. Most of our patients presented with a mild disease according to MERI, while 5 (38.4%) presented with a moderate disease (Table 1).

Ten patients underwent myringoplasty. In 2 cases, middle ear manipulations were required that included granulation tissue removal in one case and tear of an adhesion and the use of a silastic sheet in another. One case underwent tympanomastoidectomy.

The mean follow-up period was 5 months. All perforations were found to be closed regardless of their size, but one (92.3% success rate). In the latter case, the patient was found to have mastoid sclerosis on CT scan, having undergone tympanoplasty 13 years earlier. The mean preoperative pure-tone average for all patients was 40.4 dB (range 10 to 90 dB). The mean postoperative pure-tone average was 26.5 dB (range 10 to 55 dB).

## 4. Discussion

Reconstruction of the tympanic membrane aims at an intact membrane, cessation of drainage, and optimal hearing improvement. The success of tympanoplasty depends on the eradication of the disease and recreation of a healthy and aerated middle ear [8]. The insertion of membranous materials results in successful closure of the tympanic membrane in 85–90% of normally ventilated middle ears [9]. Healing has a much poorer prognosis in cases of suspected tubal dysfunction, adhesive processes, tympanic fibrosis, bilateral disease, and defects of the entire tympanic membrane [10–12]. 

In our study, we modified the known postauricular tympanoplasty technique in order to gain higher success rates in cases with anterior or total/subtotal perforations, in which higher failure rates are reported. Various surgical techniques for closure of anterior perforation have been reported [4–7] demonstrating higher rates of perforation closure compared to the traditional technique (Table 2). Most of these techniques include various combinations of overlay and underlay techniques, with or without raising the anterior aspect of the annulus for better anchoring of the graft. The use of more than one technique might imply the need for a more experienced surgeon for closure of the anterior part of the tympanic membrane.

In this prospective study, we present our experience with a modification of the traditional inferior tympanoplasty technique. Our cohort presented with poor prognostic factors—all had involvement of the anterior portion of the tympanic membrane; 50% of our patients have had chronic otitis media of one or both ears and half of these had undergone middle ear surgery in the ear operated upon in our study. In 2 cases, the CT scan demonstrated middle ear pathology including mastoid sclerosis in 1 case. Furthermore, half of the cases presented with either marginal, subtotal, or total perforations, which are known to have a worse outcome whilst utilizing the standard postauricular tympanoplasty technique. Furthermore, the MERI of 5 (38.4%) of our patients was equal to moderate disease.

Notwithstanding all the above, we achieved a high closure success rate in our study. This high success rate is attributed to the fact that our technique provides the surgeon with better access to the anterior part of the tympanic membrane and superior management of the graft. Our technique brings the surgeon closer to the pathology, bypassing middle ear obstacles such as the chorda tympany and the malleus long process, and therefore requires shorter grafts. Treatment of periossicular disease is also feasible while using this technique as middle ear access is good and the surgeon is less concerned by the shorter graft when performing ossiculoplasty. Furthermore, the technique uses the same principals as the standard posterior tympanoplasty method, does not require special skills or a very experienced surgeon, and a very short learning curve is expected. No specific complications were encountered while using this modification of the traditional approach. Therefore, we would recommend utilizing inferior tympanoplasty in all cases requiring closure of an anterior, subtotal, or total tympanic membrane perforations. 

## 5. Conclusion

The inferior tympanoplasty technique provides a favorable outcome in terms of tympanic membrane closure and hearing improvement for anterior perforations, even in difficult and complex cases, is based on a well-known technique, and is easy to implement.

## Figures and Tables

**Table 1:** 

No	Age	Sex	Side	Perforation location	Imaging	Comorbidity	MERI
1	12	M	Right	Central superior anterior	Normal		2
2	69	F	Left	Marginal anterior inferior	Left mastoid air cells effusion	HTN, hypercholesterolemia	3
3	16	F	Right	Central anterior	NA	s/p right and left tympanoplasty	5
4	61	M	Right	Marginal anterior	NA	IHD, HTN, and s/p left tympanoplasty 1970	4
5	24	F	Right	Total	NA		1
6	16	M	Right	Central anterior	NA		1
7	34	M	Left	Central anterior	Post right mastoidectomy, normal left ear	s/p right mastoidectomy	2
8	48	F	Right	Central anterior	Right mastoid sclerosis	s/p right tympanoplasty	5
9	13	F	Right	Marginal anterior	NA		1
10	48	F	Left	Subtotal	NA	Right ear perforation	1
11	19	M	Left	Central anterior inferior	NA	s/p right tympanoplasty	4
12	54	F	Right	Central anterior inferior	NA	IHD, HTN	1
13	20	F	Left	Total	NA	s/p left tympanoplasty	5

IDH: ischemic heart disease; HTN: hypertension; s/p: status post; MERI: Middle Ear Risk Index.

**Table 2:** 

Study	Hearing improvement (db)	Perforation closure (%)	Remarks
Jung and Park [7]	N/A	97	N/A
Lee et al. [4]	15	98.8	N/A
Kartush et al. [6]	5.6	100	10% late perforation
